# ATP stimulates appetite by enhancing the expression of hypothalamic orexigenic neuropeptides

**DOI:** 10.1186/s13041-025-01220-y

**Published:** 2025-06-10

**Authors:** Nayoun Kim, Eun-Kyoung Kim

**Affiliations:** 1 Department of Brain Sciences, Daegu Gyeongbuk Institute of Science and Technology, Daegu, Republic of Korea; 2 Neurometabolomics Research Center, Daegu Gyeongbuk Institute of Science and Technology, Daegu, Republic of Korea

**Keywords:** Hypothalamus, Appetite, AGRP/NPY neuron, ATP, P2X4R

## Abstract

Hypothalamic neuropeptides play a pivotal role in regulating appetite and energy homeostasis. Extracellular ATP, a key signaling molecule in the hypothalamus, is associated with neuronal activity and metabolic processes. However, its role in appetite control remains unclear. This study explored how sustained extracellular ATP regulates the expression of hypothalamic orexigenic neuropeptides *Agrp* and *Npy*. The administration of ATP alone reduced food intake, body weight, and orexigenic neuropeptide expression in mice. Conversely, inhibition of ATP conversion into AMP using the ectonucleoside triphosphate diphosphohydrolase inhibitor ARL67156 caused a transient increase in these parameters. Prolonged extracellular ATP was shown to upregulate *Agrp* and *Npy* expression via purinergic P2X4 receptor (P2X4R) activation in AGRP/NPY-expressing cells. Activation of P2X4R induced CaMKII phosphorylation, which subsequently led to CREB phosphorylation and upregulation of orexigenic neuropeptides. Our findings reveal a mechanism whereby extracellular ATP accumulation promotes appetite through P2X4R-CaMKII-CREB signaling, shedding light on how extracellular ATP impacts hypothalamic appetite control.

**Supplementary Information:**

The online version contains supplementary material available at 10.1186/s13041-025-01220-y.

## Main text

Hypothalamic arcuate nucleus (ARC), which includes AGRP/NPY and POMC/CART neurons, plays a central role in maintaining homeostasis by integrating metabolic and physiological signals to regulate energy balance and neuroendocrine functions [1, 2]. Among these signals, extracellular ATP acts as a transmitter that regulates neuronal activity and metabolic processes [3, 4]. Extracellular ATP is rapidly hydrolyzed by ectonucleotidases, such as ectonucleoside triphosphate diphosphohydrolase (ENTPD) and ecto-5′-nucleotidase, producing adenosine [5], which has been shown to inhibit AGRP neuron activity [6]. However, when ectonucleotidase function is impaired, extracellular ATP levels remain elevated, leading to reduced adenosine concentrations. This dynamic interplay between ATP and adenosine is believed to play a critical role in regulating appetite and maintaining energy homeostasis.

A previous study reported that optogenetic activation of hypothalamic tanycytes through Ca^2+^-permeable channelrhodopsin increases intracellular Ca^2+^ levels in tanycytes, leading to ATP release. This ATP subsequently depolarizes ARC neurons ex vivo and triggers a transient rise in food intake in vivo [7]. Conversely, blocking purinergic receptors or reducing Ca^2+^ signaling in tanycytes inhibited the activation of ARC neurons [7]. These findings indicate that extracellular ATP transiently modulates neuronal activity via purinergic signaling, resulting in a short-term change in food intake. However, the precise signaling pathways underlying the orexigenic effects of ATP remain unclear. In this study, we aim to investigate how the accumulation of extracellular ATP regulates the expression of orexigenic neuropeptides *Agrp* and *Npy* that stimulate food intake. By elucidating these mechanisms, this study may provide deeper insights into the role of ATP in appetite control in hypothalamic neurons.

**Fig. 1:**
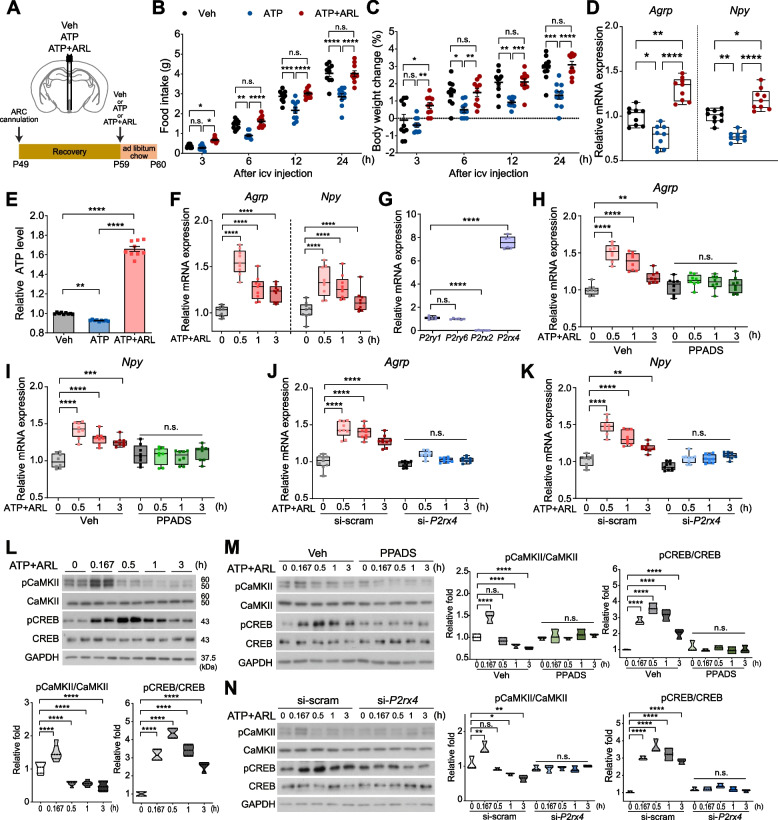
ATP induces upregulation of *Agrp* and *Npy* expression via P2X4 receptors. **A** The schematic strategy and schedule of Veh, 0.2 nmol ATP or 0.2 nmol ATP + 0.2 nmol ARL67156 (ATP + ARL) into the ARC through an implanted cannula. **B, C** Food intake (B) and body weight change (C) for 24 h after icv injection (*n* = 10 per group). **D** Relative *Agrp* and *Npy* expression levels in mice 4 h after icv injection (*n* = 9 per group). **E** Relative extracellular ATP levels in N41 cells with treatment of Veh, 0.1 mM ATP or 0.1 mM ATP + 0.1 mM ARL67156 (ATP + ARL) for 3 h (*n* = 9 per group). **F** Time courses of relative *Agrp* and *Npy* expression levels in N41 cells with ATP + ARL (*n* = 9 per group). **G** Relative mRNA levels of purinergic receptors in N41 cells (*n* = 6). **H**, **I** Time courses of relative *Agrp* (H) and *Npy* (I) expression levels in N41 cells treated with ATP + ARL in the presence or absence of 0.01 mM PPADS (P2XR antagonist) (*n* = 8 per group). **J**, **K** Time courses of relative *Agrp* (J) and *Npy* (K) expression levels in N41 cells transfected with si-scram or si-*P2rx4* and treated with ATP + ARL (*n* = 9 per group). **L**‒**N** Western blot analysis of phosphorylation of CaMKII and CREB after ATP + ARL treatment of N41 cells for 3 h (L) (*n* = 4 per group), ATP + ARL treatment of N41 cells for 3 h in the presence or absence of PPADS (M) (*n* = 3 per group) and ATP + ARL treatment of si-scram or si-*P2rx4* N41 cells for 3 h (N) (*n* = 3 per group). Data are mean ± s.e.m., boxes indicating the interquartile range with whiskers or truncated violin plots. Significance was determined by two-way ANOVA with Tukey’s multiple-comparisons test in **(B**‒**E)** and otherwise one-way ANOVA with Dunnett’s multiple comparisons test. *P < 0.5, **P < 0.01, ***P < 0.001, ****P < 0.0001. n.s., not significant

To investigate the role of ATP in regulating appetite through the hypothalamic ARC, we administered ATP alone or in combination with ARL67156, an ENTPD inhibitor (ATP + ARL) to the ARC in mice (Fig. 1A). Administration of ATP alone significantly decreased food intake and body weight after 6 h, and this reduction persisted up to 24 h. In contrast, ATP + ARL caused a transient increase in food intake and body weight at 3 h compared to vehicle (Veh) injection, but these effects were no longer observed after 6 h (Fig. 1B, C). Notably, the difference in the effects of ATP and ATP + ARL administration was sustained throughout 24 h. Consistently, ATP alone reduced mRNA expression of *Agrp* and *Npy*, whereas ATP + ARL increased their expression (Fig. 1D). These results suggest that ATP alone suppresses orexigenic neuropeptide expression and appetite, whereas sustained ATP (i.e., ATP + ARL) enhances them.

To clarify the orexigenic role of sustained ATP in AGRP/NPY neurons, we measured extracellular ATP levels in AGRP/NPY-expressing neuronal cells (mHypoE-N41, hereafter referred to as N41) following treatment with ATP alone or ATP + ARL. Extracellular ATP levels decreased when treated with ATP alone, whereas co-treatment with ATP + ARL resulted in increased ATP levels (Fig. 1E). These findings suggest that ATP, in the absence of ARL, is metabolized into compounds such as ADP, AMP, or adenosine, while ATP + ARL preserves ATP levels. We next examined the mRNA expression of *Agrp* and *Npy* under ATP + ARL conditions over a 3-h period. The results revealed a significant increase in the expression of these genes in N41 cells (Fig. 1F).

AGRP/NPY neurons are known to express purinergic receptors including P2X4R and P2Y6R [8–10]. ATP is the agonist for P2XR and P2YR families [11]. Subsequent screening of ATP-binding receptors revealed that *P2rx4* mRNA was the predominant purinergic receptor candidate in N41 cells (Fig. 1G). Under control conditions, including si-scram and Veh treatment, ATP + ARL significantly increased *Agrp* and *Npy* expression. However, these increases were mitigated by pyridoxalphosphate-6-azophenyl-2',4'-disulfonic acid (PPADS), a pharmacological inhibitor of P2X4R (Fig. 1H, I), or through genetic knockdown of *P2rx4* (Fig. 1J, K). These results suggest that elevated extracellular ATP regulates the expression of *Agrp* and *Npy* via P2X4R.

Extracellular ATP binding to P2XRs triggers Ca^2+^ influx, leading to the phosphorylation and activation of calcium/calmodulin-dependent kinase II (CaMKII) [12–14]. To explore whether CaMKII functions as downstream of ATP–P2X4R signaling in regulating *Agrp* and *Npy* expression, we analyzed the phosphorylation dynamics of CaMKII and cAMP response element–binding protein (CREB) in ATP + ARL-treated N41 cells. The results showed that CaMKII phosphorylation peaked at 0.167 h, while CREB phosphorylation remained elevated until 0.5 h (Fig. 1L). Notably, pharmacological and genetic inhibition of P2X4R completely blocked the ATP + ARL-induced increase in phosphorylation levels of both CaMKII and CREB (Fig. 1M, N). These findings suggest that extracellular ATP engages P2X4R, initiating CaMKII phosphorylation, which subsequently drives CREB phosphorylation and enhances the expression of *Agrp* and *Npy*.

ATP induces activation of P2X4R on AGRP/NPY neurons, promoting the release of GABA onto POMC neurons [8]. Unlike P2XRs, which function as ligand-gated ion channels, P2YRs are G-protein-coupled receptors that exhibit higher sensitivity to ADP or UDP than to ATP. P2YRs play significant roles in food intake and insulin sensitivity [9, 10, 15]. Specifically, the phosphorylation of ERK by UDP-activated P2Y6R on AGRP/NPY neurons, but not on POMC neurons, stimulates food intake [9]. However, the influence of P2X4R on modulating appetite-related neuropeptides has not been documented. In our study, P2X4R was found to regulate the expression of *Agrp* and *Npy* in N41 cells, particularly in the presence of ARL67156 which inhibits the conversion of extracellular ATP.

We propose that ATP signals CaMKII phosphorylation through P2X4R, leading to CREB phosphorylation and the subsequent upregulation of *Agrp* and *Npy* expression. Our data demonstrate that inhibiting ATP conversion with ARL67156 enhances *Agrp* and *Npy* expression. Under physiological conditions, ATP is rapidly converted into adenosine, which exerts anorexigenic effects [16]. However, this conversion is impaired in pathological states characterized by dysregulated ENTPD expression, such as autoimmune diseases, resulting in sustained extracellular ATP accumulation [17]. While current evidence primarily focuses on the roles of ENTPD in immune cells and vascular endothelial cells [18], further investigations are required to unravel the precise mechanisms by which ATP and adenosine dynamics influence hypothalamic neuronal signaling and appetite regulation under various pathological conditions. Gaining a deeper understanding of these processes could offer valuable insights into the interplay between ATP and adenosine in regulating energy homeostasis, which may open new therapeutic strategies to treat metabolic diseases such as obesity and diabetes.

## Supplementary Information

Additional file 1

## Data Availability

No datasets were generated or analysed during the current study.

## Abbreviations

ARC: Arcuate nucleus
ATP + ARL: Co-treatment of ATP with ARL67156
CaMKII: Calcium/calmodulin-dependent kinase II
CREB: cAMP response element–binding protein
ENTPD: Ectonucleoside triphosphate diphosphohydrolase
P2X4R: P2X4 receptor
PPADS: Pyridoxalphosphate-6-azophenyl-2',4'-disulfonic acid
Veh: Vehicle
