# Supplementary material for: ATP stimulates appetite by enhancing the expression of hypothalamic orexigenic neuropeptides
Source: Mol Brain. 2025 Jun 10;18:49. doi: 10.1186/s13041-025-01220-y (PMC12150506; doi:10.1186/s13041-025-01220-y)
Supplement: Supplementary file 1 — Additional file 1 [file 13041_2025_1220_MOESM1_ESM.docx]

**Materials and Methods**

**Animals**

Mice (C57BL6/N, KOATECH) were housed under specific pathogen–free conditions in a temperature-controlled room (20–26 °C) with 40–60% humidity under a 12 h light/dark cycle in individually ventilated cages. Mice were fed an ad libitum chow diet (LabDiet) containing 62% of calories from carbohydrates, 24% from protein, and 13% from fat.

**Measurements of food intake and body weight**

Food intake and body weight were measured daily at the onset of the dark cycle in individually housed mice. Following the intracerebroventricular (icv) injection of vehicle (Veh), ATP, or ARL67156 at the onset of the dark cycle, food intake and body weight were monitored at 3, 6, 12, and 24 h post-injection.

**Cannulation and icv injection**

For icv injection, all mice were anesthetized via intraperitoneal (ip) injection of a mixture of Zoletil 50 (30 mg/kg; Virbac) and Rompun (10 mg/kg; Bayer Korea). A bilateral cannula (PlasticsOne) was implanted at the ARC (1.3 mm posterior, ±0.5 mm lateral, and 5.6mm ventral to bregma) for the administration of ATP (0.2 nmol; Sigma-Aldrich), ARL67156 (0.2 nmol; Tocris), or saline as the Veh. Following the cannula implantation, the scalp was sealed with dental cement (Dentsply), and mice were housed in individual cages to recover for 10 days.

**Cell culture**

The mHypoE-N41 (CLU121) cell line was purchased from Cellutions Biosystems Inc. and maintained in DMEM culture medium (Sigma-Aldrich) supplemented with 10 % heat-inactivated fetal bovine serum (Hyclone Laboratories) and 1% penicillin-streptomycin (Hyclone Laboratories). The fetal bovine serum was heat-inactivated at 55 °C.

**ATP assay**

Extracellular ATP levels were determined by the ATP Bioluminescence Assay Kit HS II (Roche) following the manufacturer’s instructions. The media were collected to measure extracellular ATP levels. The collected extracellular media were mixed with luciferase reagent, and luminescence was detected using a SpectraMax-L microplate reader (Molecular Devices). Cells were lysed in TE buffer, heated at 95 °C for 7 min, and centrifuged (21,130 ×*g*) for 3 min at 4 °C. ATP levels were normalized to the amount of total protein quantified using a BCA assay kit (Thermo Fisher Scientific) in cell lysates.

**Drug treatments**

N41 cells were treated with ATP (0.1 mM), ARL67156 (0.1 mM), or PPADS (0.01 mM; Tocris) using sterilized water as the Veh. All concentrations of drugs applied to cell lines were within the range that maintained cell viability.

**Transfection of small interfering RNA**

Cells were transfected with *P2rx4* siRNA (100 nM) using Lipofectamine 3000 (Life Technologies) following the manufacturer’s instructions. *P2rx4* siRNA was SMARTpools produced by Dharmacon. Scrambled siRNA (100 nM; ON-TARGETplus Non-targeting Pool) from Dharmacon was used as a transfection control.

**Real-time qPCR**

Samples were prepared as previously described [1]. Real-time qPCR was performed using CFX96™ Real-Time PCR (Bio-Rad). Primers were synthesized by Bionics (Supplementary Table 1). Reaction mixture contained 3 μl of cDNA template (1 μg/μl), 10 μl of TB Green PCR master mix (TaKaRa Biotechnology), and 5 pmol of each gene-specific primer in a total volume of 20 μl. The cDNA was denatured at 95 °C for 1 min, followed by 40 cycles of PCR at 95 °C for 10 s, and 60 °C for 30 s. Gene expression levels were analyzed using ∆∆Cq method with *Gapdh* expression as the control. The qPCR experiments included three sets of biological replicates, each comprising an average of three technical replicates.

**Western blot**

Cells were lysed as previously reported [2]. Protein concentrations in the lysates were measured with a BCA assay kit using a VersaMax microplate reader (Molecular Devices). Equal amounts of protein (9 μg) were resolved by SDS-PAGE and transferred to Immobilon-P membrane (Merck). The membranes were blocked by 5% skim milk in TBST (20 mM Tris, 125 mM NaCl, 0.1% Tween 20, pH 7.4) for 1 h. Subsequently, the membranes were incubated with the primary antibody for 2 h at room temperature or overnight at 4 °C. After washing, the membranes were incubated with horseradish peroxidase–conjugated secondary antibody for 1 h at room temperature. Chemiluminescent signals were developed using SuperSignal West Pico Chemiluminescent Substrate (Thermo Fisher Scientific) and quantified using Image J software (National Institutes of Health). All antibodies were purchased from Cell Signaling Technology: phospho-CREB (Ser133), CREB, phospho-CaMKII (Thr286), CaMKII and GAPDH.

**Statistical analysis**

Data were presented as mean ± s.e.m., box plots indicating the interquartile range with whiskers extending to the highest and lowest values, or truncated violin plots. All statistical analyses were performed using GraphPad Prism 9.1.1 (GraphPad Software Inc.). Statistical significance was determined by one-way and two-way ANOVA for comparisons across multiple groups. *P-*values of less than 0.05 were considered statistically significant. The *p-*values for each result are provided in Supplementary Table 2.

**Supplementary Table 1. The sequence of primers for qRT-PCR**

| **Gene** | **Forward primer sequence (5′ to 3′)** | **Reverse primer sequence (5′ to 3′)** |
| --- | --- | --- |
| *Agrp* | CTG CAG ACC GAG CAG AAG A | TGC GAC TAC AGA GGT TCG TG |
| *Npy* | CAG AAA ACG CCC CCA GAA | AAA AGT CGG GAG AAC AAG TTT CAT T |
| *Gapdh* | ATC ACT GCC ACC CAG AAG AC | ACA CAT TGG GGG TAG GAA CA |
| *P2rx2* | GCT GCT CAT TCT GCT TTA CTT CG | TCC CAC ACT TTG TGT TCC GA |
| *P2rx4* | AGA CGG ACC AGT GAT GCC TAA C | TGG AGT GGA GAC CGA GTG AGA |
| *P2ry1* | TCG TGT CTC CAT TCT GCT TG | CGA CAG GGT TTA TGC CAC TT |
| *P2ry6* | GGG TAG TGT GTG GAG TCG TG | AGC GAG TAG ACA GGA TGG GT |

**Supplementary Table 2. *P*-values for statistical comparisons in Figure 1**

| Figure 1B |  | Figure 1C |  |
| --- | --- | --- | --- |
| 3 h | ***P-*value** | 3 h | ***P-*value** |
| **Veh** vs. **ATP** | 0.7625 | **Veh** vs. **ATP** | 0.5814 |
| **Veh** vs. **ATP+ARL** | 0.0254 | **Veh** vs. **ATP+ARL** | 0.0141 |
| **ATP** vs. **ATP+ARL** | 0.0236 | **ATP** vs. **ATP+ARL** | 0.0036 |
| 6 h |  | 6 h |  |
| **Veh** vs. **ATP** | 0.0043 | **Veh** vs. **ATP** | 0.0135 |
| **Veh** vs. **ATP+ARL** | 0.5016 | **Veh** vs. **ATP+ARL** | 0.995 |
| **ATP** vs. **ATP+ARL** | <0.0001 | **ATP** vs. **ATP+ARL** | 0.0026 |
| 12 h |  | 12 h |  |
| **Veh** vs. **ATP** | 0.0003 | **Veh** vs. **ATP** | 0.0014 |
| **Veh** vs. **ATP+ARL** | 0.6333 | **Veh** vs. **ATP+ARL** | 0.9986 |
| **ATP** vs. **ATP+ARL** | <0.0001 | **ATP** vs. **ATP+ARL** | 0.0003 |
| 24 h |  | 24 h |  |
| **Veh** vs. **ATP** | <0.0001 | **Veh** vs. **ATP** | 0.0002 |
| **Veh** vs. **ATP+ARL** | 0.9973 | **Veh** vs. **ATP+ARL** | 0.8086 |
| **ATP** vs. **ATP+ARL** | <0.0001 | **ATP** vs. **ATP+ARL** | <0.0001 |
| Figure 1D |  |  |  |
| *Agrp* | ***P-*value** | *Npy* | ***P-*value** |
| **Veh**vs.**ATP** | 0.0178 | **Veh**vs.**ATP** | 0.003 |
| **Veh**vs.**ATP+ ARL** | 0.0027 | **Veh**vs.**ATP+ ARL** | 0.0106 |
| **ATP**vs.**ATP+ ARL** | <0.0001 | **ATP**vs.**ATP+ ARL** | <0.0001 |
| Figure 1E |  |  |  |
|  | ***P-*value** |  |  |
| **Veh**vs.**ATP** | 0.0064 |  |  |
| **Veh**vs.**ATP+ ARL** | <0.0001 |  |  |
| **ATP**vs.**ATP+ ARL** | <0.0001 |  |  |
| Figure 1F |  |  |  |
| *Agrp* | ***P-*value** | *Npy* | ***P-*value** |
| **0 h** vs. **0.5 h** | <0.0001 | **0 h** vs. **0.5 h** | <0.0001 |
| **0 h** vs. **1 h** | <0.0001 | **0 h** vs. **1 h** | <0.0001 |
| **0 h** vs. **3 h** | <0.0001 | **0 h** vs. **3 h** | <0.0001 |
| Figure 1G |  |  |  |
|  | ***P-*value** |  |  |
| ***P2ry1*** vs. ***P2ry6*** | 0.9424 |  |  |
| ***P2ry1*** vs. ***P2rx2*** | <0.0001 |  |  |
| ***P2ry1*** vs. ***P2rx4*** | <0.0001 |  |  |
| Figure 1H |  | Figure 1I |  |
| *Agrp* | ***P-*value** | *Npy* | ***P-*value** |
| **Veh 0 h** vs. **Veh 0.5 h** | <0.0001 | **Veh 0 h** vs. **Veh 0.5 h** | <0.0001 |
| **Veh 0 h** vs. **Veh 1 h** | <0.0001 | **Veh 0 h** vs. **Veh 1 h** | <0.0001 |
| **Veh 0 h** vs. **Veh 3 h** | 0.0072 | **Veh 0 h** vs. **Veh 3 h** | 0.0005 |
| **Veh 0 h** vs. **PPADS 0 h** | 0.8468 | **Veh 0 h** vs. **PPADS 0 h** | 0.61 |
| **Veh 0 h** vs. **PPADS 0.5 h** | 0.704 | **Veh 0 h** vs. **PPADS 0.5 h** | 0.8533 |
| **Veh 0 h** vs. **PPADS 1 h** | 0.5398 | **Veh 0 h** vs. **PPADS 1 h** | 0.8489 |
| **Veh 0 h** vs. **PPADS 3 h** | 0.655 | **Veh 0 h** vs. **PPADS 3 h** | 0.7023 |
| Figure 1J |  | Figure 1K |  |
| *Agrp* | ***P-*value** | *Npy* | ***P-*value** |
| **scram 0 h** vs.**scram 0.5 h** | <0.0001 | **scram 0 h** vs.**scram 0.5 h** | <0.0001 |
| **scram 0 h** vs. **scram 1 h** | <0.0001 | **scram 0 h** vs. **scram 1 h** | <0.0001 |
| **scram 0 h** vs. **scram 3 h** | <0.0001 | **scram 0 h** vs. **scram 3 h** | 0.0045 |
| **scram 0 h** vs. ***P2rx4* 0 h** | 0.904 | **scram 0 h** vs. ***P2rx4* 0 h** | 0.81 |
| **scram 0 h** vs. ***P2rx4* 0.5 h** | 0.823 | **scram 0 h** vs. ***P2rx4* 0.5 h** | 0.5533 |
| **scram 0 h** vs. ***P2rx4* 1 h** | 0.9564 | **scram 0 h** vs. ***P2rx4* 1 h** | 0.8914 |
| **scram 0 h** vs. ***P2rx4* 3 h** | 0.9444 | **scram 0 h** vs. ***P2rx4* 3 h** | 0.7672 |
| Figure 1L |  |  |  |
| pCaMKII/CaMKII | ***P-*value** | pCREB/CREB | ***P-*value** |
| **0 h** vs. **0.167 h** | <0.0001 | **0 h** vs. **0.167 h** | <0.0001 |
| **0 h** vs. **0.5 h** | <0.0001 | **0 h** vs. **0.5 h** | <0.0001 |
| **0 h**vs. **1 h** | <0.0001 | **0 h**vs. **1 h** | <0.0001 |
| **0 h** vs. **3 h** | <0.0001 | **0 h** vs. **3 h** | <0.0001 |
| Figure 1M |  |  |  |
| pCaMKII/CaMKII | ***P-*value** | pCREB/CREB | ***P-*value** |
| **Veh 0 h** vs. **Veh 0.167 h** | <0.0001 | **Veh 0 h** vs. **Veh 0.167 h** | <0.0001 |
| **Veh 0 h** vs. **Veh 0.5 h** | 0.8013 | **Veh 0 h** vs. **Veh 0.5 h** | <0.0001 |
| **Veh 0 h**vs. **Veh 1 h** | <0.0001 | **Veh 0 h**vs. **Veh 1 h** | <0.0001 |
| **Veh 0 h** vs. **Veh 3 h** | <0.0001 | **Veh 0 h** vs. **Veh 3 h** | <0.0001 |
| **Veh 0 h** vs. **PPADS 0 h** | 0.9999 | **Veh 0 h** vs. **PPADS 0 h** | 0.9695 |
| **Veh 0 h** vs. **PPADS 0.167 h** | >0.9999 | **Veh 0 h** vs. **PPADS 0.167 h** | >0.9999 |
| **Veh 0 h** vs. **PPADS 0.5 h** | >0.9999 | **Veh 0 h** vs. **PPADS 0.5 h** | 0.9977 |
| **Veh 0 h**vs. **PPADS 1 h** | 0.9517 | **Veh 0 h**vs. **PPADS 1 h** | >0.9999 |
| **Veh 0 h** vs. **PPADS 3 h** | 0.9952 | **Veh 0 h** vs. **PPADS 3 h** | >0.9999 |
| Figure 1N |  |  |  |
| pCaMKII/CaMKII | ***P-*value** | pCREB/CREB | ***P-*value** |
| **Veh 0 h** vs. **Veh 0.167 h** | 0.0012 | **Veh 0 h** vs. **Veh 0.167 h** | <0.0001 |
| **Veh 0 h** vs. **Veh 0.5 h** | 0.8922 | **Veh 0 h** vs. **Veh 0.5 h** | <0.0001 |
| **Veh 0 h**vs. **Veh 1 h** | 0.4256 | **Veh 0 h**vs. **Veh 1 h** | <0.0001 |
| **Veh 0 h** vs. **Veh 3 h** | 0.0021 | **Veh 0 h** vs. **Veh 3 h** | <0.0001 |
| **Veh 0 h** vs. **PPADS 0 h** | 0.9963 | **Veh 0 h** vs. **PPADS 0 h** | 0.8971 |
| **Veh 0 h** vs. **PPADS 0.167 h** | >0.9999 | **Veh 0 h** vs. **PPADS 0.167 h** | 0.8438 |
| **Veh 0 h** vs. **PPADS 0.5 h** | 0.9979 | **Veh 0 h** vs. **PPADS 0.5 h** | 0.9939 |
| **Veh 0 h**vs. **PPADS 1 h** | >0.9999 | **Veh 0 h**vs. **PPADS 1 h** | 0.9959 |
| **Veh 0 h** vs. **PPADS 3 h** | >0.9999 | **Veh 0 h** vs. **PPADS 3 h** | >0.9999 |

**References for supplementary materials and methods**

1. Kim S, Kim N, Park S, Jeon Y, Lee J, Yoo SJ, et al. Tanycytic TSPO inhibition induces lipophagy to regulate lipid metabolism and improve energy balance. Autophagy. 2020;16(7):1200-20.

2. Park S, Oh S, Kim N, Kim EK. HMBA ameliorates obesity by MYH9- and ACTG1-dependent regulation of hypothalamic neuropeptides. EMBO Mol Med. 2023;15(12):e18024.
